# Higher‐resolution quantification of white matter hypointensities by large‐scale transfer learning from 2D images on the JPSC‐AD cohort

**DOI:** 10.1002/hbm.25899

**Published:** 2022-05-07

**Authors:** Benjamin Thyreau, Yasuko Tatewaki, Liying Chen, Yuji Takano, Naoki Hirabayashi, Yoshihiko Furuta, Jun Hata, Shigeyuki Nakaji, Tetsuya Maeda, Moeko Noguchi‐Shinohara, Masaru Mimura, Kenji Nakashima, Takaaki Mori, Minoru Takebayashi, Toshiharu Ninomiya, Yasuyuki Taki, Japan Prospective Studies Collaboration for Aging and Dementia (JPSC‐AD) Study Group

**Affiliations:** ^1^ Smart‐Aging Research Center, Institute of Development, Aging, and Cancer Tohoku University Sendai Japan; ^2^ Department of Aging Research and Geriatric Medicine, Institute of Development, Aging, and Cancer Tohoku University Sendai Japan; ^3^ Department of Geriatric Medicine and Neuroimaging Tohoku University Hospital Sendai Japan; ^4^ Department of Psychological Sciences University of Human Environments Matsuyama Japan; ^5^ Department of Epidemiology and Public Health, Graduate School of Medical Sciences Kyushu University Fukuoka Japan; ^6^ Department of Social Medicine, Graduate School of Medicine Hirosaki University Hirosaki Japan; ^7^ Division of Neurology and Gerontology, Department of Internal Medicine, School of Medicine Iwate Medical University Iwate Japan; ^8^ Department of Neurology and Neurobiology of Aging, Kanazawa University Graduate School of Medical Sciences Kanazawa University Kanazawa Japan; ^9^ Keio University School of Medicine Tokyo Japan; ^10^ National Hospital Organization, Matsue Medical Center Shimane Japan; ^11^ Department of Neuropsychiatry, Ehime University Graduate School of Medicine Ehime University Ehime Japan; ^12^ Faculty of Life Sciences, Department of Neuropsychiatry Kumamoto University Kumamoto Japan

## Abstract

White matter lesions (WML) commonly occur in older brains and are quantifiable on MRI, often used as a biomarker in Aging research. Although algorithms are regularly proposed that identify these lesions from T2‐fluid‐attenuated inversion recovery (FLAIR) sequences, none so far can estimate lesions directly from T1‐weighted images with acceptable accuracy. Since 3D T1 is a polyvalent and higher‐resolution sequence, it could be beneficial to obtain the distribution of WML directly from it. However a serious difficulty, both for algorithms and human, can be found in the ambiguities of brain signal intensity in T1 images. This manuscript shows that a cross‐domain ConvNet (Convolutional Neural Network) approach can help solve this problem. Still, this is non‐trivial, as it would appear to require a large and varied dataset (for robustness) labelled at the same high resolution (for spatial accuracy). Instead, our model was taught from two‐dimensional FLAIR images with a loss function designed to handle the super‐resolution need. And crucially, we leveraged a very large training set for this task, the recently assembled, multi‐sites Japan Prospective Studies Collaboration for Aging and Dementia (JPSC‐AD) cohort. We describe the two‐step procedure that we followed to handle such a large number of imperfectly labeled samples. A large‐scale accuracy evaluation conducted against FreeSurfer 7, and a further visual expert rating revealed that WML segmentation from our ConvNet was consistently better. Finally, we made a directly usable software program based on that trained ConvNet model, available at https://github.com/bthyreau/deep-T1-WMH.

## Graphical abstract

It is possible to identify White Matter Lesions on the high‐resolution, morphometry‐friendly, T1‐weigthed sequence directly with good accuracy. We leverage a new, large, Eldery brain cohort to train and evaluate a ConvNet identification model, solving various specific technical challenge. Two trained Radiologists judged the outcome much above FreeSurfer and even FLAIR‐based segmentation.

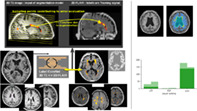

## 1 INTRODUCTION

White matter lesions (WML) frequently occur in the brain later in life (Prins & Scheltens, 2015) and can be characterized in terms of aspect and spatial distribution. On T2‐fluid‐attenuated inversion recovery (FLAIR) MRI scans, they appear as punctuated or even patchy white matter hyperintensities (WMH) that are spatially distributed around the ventricles or extend deep into the white matter in some cases. Such lesions are also visible, albeit less prominently, as hypointense patches on T1 images.

Historically, MRI has been used to quantitatively evaluate WML for clinical and research purposes using visual rating scales, with the Fazekas scale (Fazekas et al., 1987) being one of the most employed. However, in practice, WMH rating scales are of limited use beyond a purely descriptive purpose. More recently, quantitative information has become available thanks to computerized image processing (Frey et al., 2019). These two metrics can partially concur (Cedres et al., 2020; Koikkalainen et al., 2019).

The etiology of WML can vary (Wardlaw et al., 2019) and is still not well understood (Alber et al., 2019; Debette & Markus, 2010). Large occurrences of WML in the brain have reportedly been associated with negative brain traits, such as cognitive decline, dementia, stroke, or intracranial hypertension (Atwi et al., 2018; Fazekas et al., 1993; Habes et al., 2018; Lampe et al., 2019; Ngai et al., 2007; Ni et al., 2021; Pantoni, 2010; Pozorski et al., 2019; Sarica et al., 2019; Tubi et al., 2020). However, despite these broad correlation trend reports, no definitive conclusion has been made or accepted (Rhodius‐Meester et al., 2017; Vangberg et al., 2019). A large meta‐analysis of the predictive power of several factors for dementia found either mixed or no evidence for an effect of WMH (Ansart et al., 2021).

In order to disentangle the multiple putative causative factors associated with WML, more research should be undertaken involving larger, wider cohorts and automated image analysis which is capable of efficiently and reproducibly extracting the number of characteristics of WML from images. Consequently, WMH segmentation remains a popular technical problem among the image analysis research community. A frequent approach relies on atlases and histogram clustering methods (Schirmer et al., 2019). Tools such as LST (Schmidt et al., 2012), BIANCA (Griffanti et al., 2016; Sundaresan et al., 2019), and UBO (Jiang et al., 2018) were ranked favorably in a recent comparison (Heinen et al., 2019). Moreover, deep learning methods have attracted increasing attention over the past few years. Unfortunately, (Balakrishnan et al., 2021) noted that only eight of 37 authors made their method available at all, which may explain their lack of adoption by the wider clinical community.

In this manuscript, we address the problem of WMH identification from a multi‐modality (T1‐weighted imaging) and large‐scale learning perspective. We rely on Convolutional Neural Networks (ConvNets) for image learning. Our source material comprises a very large, aggregated dataset of 7694 brain images acquired in the context of the JPSC‐AD project (Ninomiya et al., 2020), a multisite, population‐based prospective cohort initiated in 2016 and has thus far recruited over 10,000 participants. To facilitate the collaboration of hospitals, the MR‐imaging component of the project was designed with flexibility in mind, with only T1‐weighted images being the common requirement.

### 1.1 Challenges

The following challenges should be considered when performing WMH identification in the most effective way.

#### 1.1.1 Population heterogeneity

As much as possible, association studies should be founded on large samples, with enough heterogeneity to cover a wide array of brain health statuses. We believe that a large prospective research cohort is more suitable than a small, specific subset of patients. On the other hand, a larger dataset also requires a greater human annotation effort, or at least, a larger evaluation effort.

#### 1.1.2 Lesions load and localization

Apparent lesions can have different etiologies depending on their shape and location, and therefore different predictive clinical importance (Lampe et al., 2019). Periventricular (PVWM) lesions and deep‐white matter lesions (DWM), which have varying effects on cognition and arterial pressure, may be entirely different entities (Griffanti et al., 2018). Small, irregular patches may be a sign of ischemia, whereas larger, smoother periventricular lesions may simply be a benign symptom of aging. Small punctate lesions may eventually develop into pathologies, but their role still needs to be clarified (Prins & Scheltens, 2015). Local features of the vasculature may also have an impact (Debette et al., 2019). Overall, to date, the etiology of WMH is still largely unknown, and existing classification scales partly reflect this (van Straaten et al., 2006).

In the computational domain, consequently, an identification algorithm should not rely solely on blindly aggregated metrics such as total voxel overlap as its unique objective, or it will risk missing this distinction and would become biased toward large patches, ignoring the low voxel contributions of punctate lesions and leading to suboptimal clinical predictive power.

#### 1.1.3 Sequence and resolution

FLAIR is one of the optimal sequences for observing WMH, but 3D FLAIR sequences with high SNR are not yet widespread in hospital settings. Two‐dimensional FLAIR sequences are easier to obtain but suffer from poor resolution along the slice axis, which not only reduces accuracy in that dimension but also introduces a source of mismatch for longitudinal comparisons.

Alternatively, lesion‐induced WMHs are visible on T1 weighted images in most cases, where they appear as hypointense regions (and the acronym *WMH* will refer to both contrast signals from now on). It is common to use the T1‐weighted contrast for structural, high‐resolution images of the brain, for example, to allow accurate estimates of ventricle enlargement or hippocampal volume or atrophy (Nogovitsyn et al., 2019; Thyreau et al., 2018). Therefore, it is useful to obtain WMH delineation directly from regular 3D T1 images.

However, in a major difference from FLAIR, lesions‐induced WMH on T1 images appear with the same signal intensity as gray‐matter structures and would require considerably more attention from an investigator to confidently identify them by visual inspection, particularly for infra‐cortical lesions. This, however, can be accomplished using moderns algorithms.

#### 1.1.4 Robustness and consistency

The model should be able to robustly identify lesions in all patients, with a low failure or rejection rate. In technical terms of machine learning, the model should be exposed to a dataset covering large patterns of the population. When using small samples, it is tempting to emphasize pixel‐perfect metric scores that could easily be oversensitive to the particular sample or to a particular chosen threshold. Some other types of loss exist, such as perimeter loss (el Jurdi et al., 2021), but those still assume that the target, or its perimeter, is well‐defined. Therefore, while such metrics may be a perfect way to evaluate a model's ability to learn accurately, they may not necessarily be able to adequately gauge the success of the task itself.

### 1.2 Objectives

The goals of this manuscripts are as follows:

- We explore the use of transfer‐learning from 2D‐FLAIR to 3D‐T1, introducing the use of a spatially sparse loss to handle super‐resolution needs;
- We leverage a new, very‐large cohort, the JPSC‐AD cohort. We design a two‐step learning scheme to better manage the mass of data with limited human guidance;
- For validation, we run a large‐sample automated quantitative comparison against FreeSurfer (Fischl, 2012), and a relatively large (200 images) qualitative visual evaluation by experts.
- We embedded the model trained on the full dataset into an open‐source tool available for download

## 2 MATERIALS AND METHODS

### 2.1 Dataset

The MRI brain images were obtained from the Japan Prospective Studies Collaboration for Aging and Dementia (JPSC‐AD), a project which collected data from over 10,000 participants across eight sites. The strategy followed to elucidate risk factors and the etiology of dementia is described in (Ninomiya et al., 2020). The mean age of the dataset was 74.4, starting from 65, 42% was male, and 8.5% of the dataset were diagnosed with dementia. The participants all underwent an MRI exam with at least one T1‐weighted acquisition. Other sequences were collected and made available at the discretion of the participating hospital. This level of flexibility was designed to encourage hospital participation. The scanners used in these hospitals were diverse, including 1.5 T MRI (four Philips, one Hitachi, and one General‐Electric) and 3 T MRI (one GE and one Siemens) machines.

This study collected all the FLAIR acquisitions available giving a total of 7699 images from six sites. The 2D FLAIR images comprised 18–24 slices depending on the scanning site, with slice thickness ranging from 5.9 to 7.8 mm. The axial resolution ranged from 0.47 to 0.57 mm. One site performed a systematic Fazekas rating of all its FLAIR images, but this information was not directly used for the purpose of the main ConvNet development. Regarding structural imaging, 10,017 sagittal T1‐weighted images were acquired (3D MPRAGE or SPGR sequences). The voxel dimension was 1.2 mm along the sagittal axis and varied from 0.93 to 1.02 mm in the other dimensions.

### 2.2 Preprocessing

The T1 images were processed on FreeSurfer 7.1. From these, 9985 could complete a full image processing pipeline, while the remaining images were excluded due to data handling mistakes or software failures, including non‐convergence within 24 h. However, we did not attempt to visually confirm all individual results. T1 images which had no corresponding FLAIR image were of no further use to the work described here. The T1 images were registered and resampled into an MNI space clipped immediately below the temporal lobes, measuring 184 × 202 × 72 voxels of size 1 × 1 × 1.5 mm, which defines our working space for all T1‐related ConvNet inputs and outputs in the following sections.

A rigid registration matrix, from FLAIR to T1, was estimated using NiftyReg Aladin (Modat et al., 2010). All of the 7699 registrations were visually quality‐checked based on a single middle slice, after which only five further subjects were excluded.

### 2.3 ConvNet model training

**FIGURE 1:**
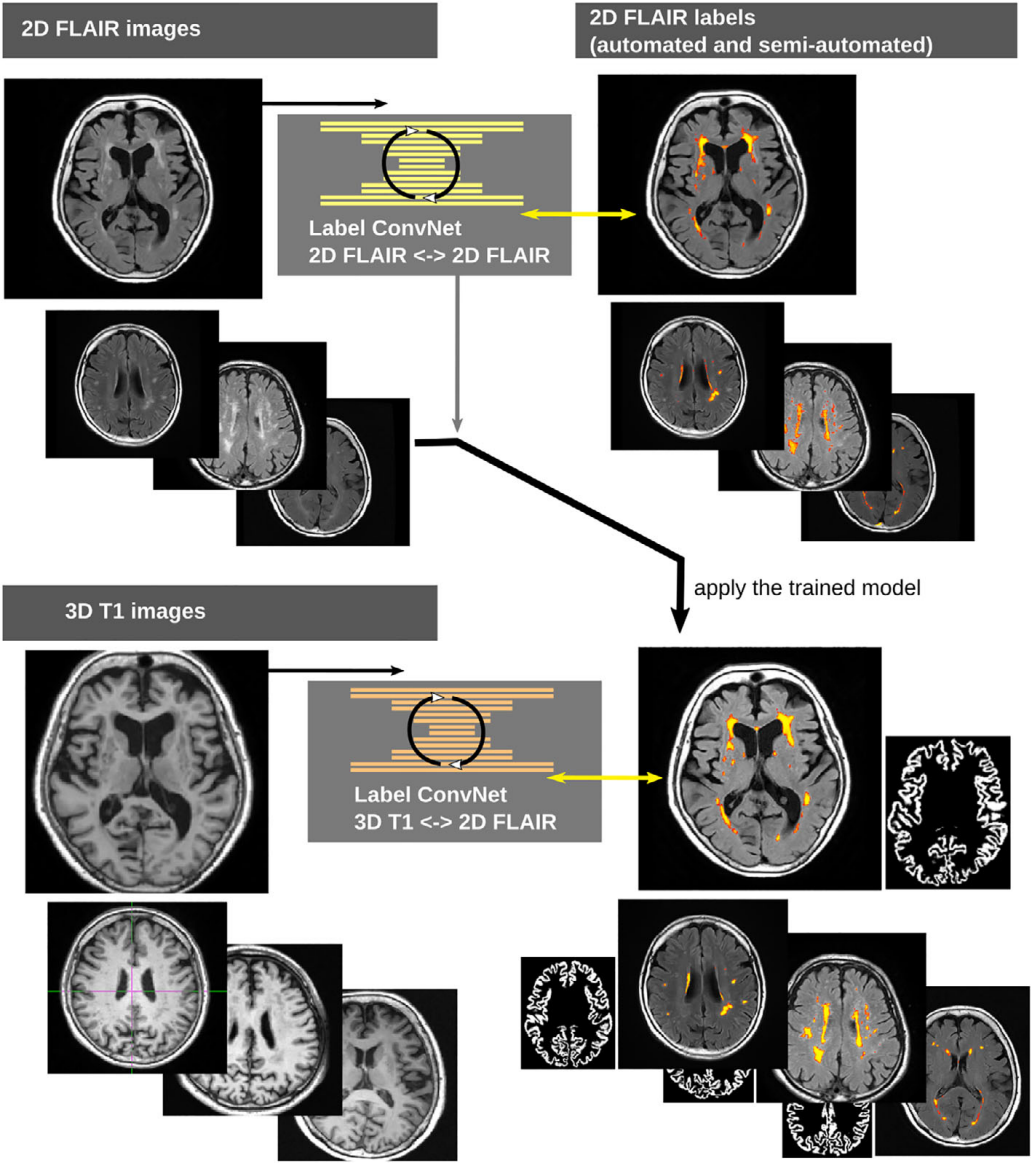
The two‐step learning process. The 2D FLAIR model (top) is trained to output a consistent FLAIR mask, initially based on the LST algorithm but with some further manual corrections. The T1 model (bottom) aims to learn a T1 WMH segmentation from those 2D‐FLAIR masks, learning across modality and resolution. It also learns a cortical ribbon as a secondary joint task. Both steps, including relevant preprocessing, use the same, single, training dataset.

The overall idea was to use the lesion information obtained from cleanly segmented 2D‐FLAIR images as targets to train a 3D‐T1 ConvNet. We achieved this by developing a two‐step process. The first step was to generate accurate labels in the 2D‐FLAIR space, and the second was to train the 3DT1 model from these sparse 2D‐FLAIR labels (Figure 1).

### 2.4 FLAIR lesion ConvNet

As a first step, the LST (Schmidt et al., 2012) FLAIR segmentation software was applied to all 2D FLAIR images. Despite LST being considered accurate and a strong performer in comparative studies (Heinen et al., 2019; Ribaldi et al., 2021; Vanderbecq et al., 2020), a small but significant fraction of the results inevitably contained some inaccurate or unsatisfactory segmentation (Figure S2). While we could simply review all images and discard these failed cases, this would risk losing some interesting corner‐cases, which would run contrary to our goal of robustness. Instead, we aimed to identify such cases and manually fix a subset of them. To achieve this, we rely on one ability of ConvNet, that is, they integrate knowledge across multiple samples and learn the main trends of the data before the particular deviations (Arpit et al., 2017). Therefore, we first trained a “FLAIR” ConvNet model to mimic the LST segmenter on a large training set and stopped the training when the loss function appeared to reach a slower convergence regime. Since the dataset is large, and the ConvNets do not easily overfit, we reasoned that we would find many unusual or mis‐segmented samples among those that exhibited a large loss error.

Half of the dataset was used to train this FLAIR ConvNet, and no test set was involved. The network had a simple U‐Net architecture, and the parameters are described in Table 1. The input format was always the native FLAIR image space, even though the number of slices varied between sites. Given its “convolutional” nature, the network naturally handles input size variations, without the need to introduce inflexible padding, or worse, resizing and resampling artifacts. Consequently, for practical simplicity, the mini‐batch size was set to 1 during the optimization, and batch‐normalizations were avoided in favor of instance‐normalizations in the network. Geometrical augmentation was performed with random rotations—only around the axial axis, to avoid inter‐slice resampling—and random translations.

**TABLE 1:** Topology of the ConvNets used through this study.

T1‐WMH net	T1‐ROI net	FLAIR net (WMH or ROI)
Input: a T1 image in the MNI workspace Conv3(1, 24), InstNorm, ReLU, Conv3(24, 64), ReLU (block0) MaxPool Conv3(64, 64), InstNorm, ReLU, Conv3(64, 64), ReLU (block1) MaxPool Conv3(64, 64), InstNorm, ReLU, Conv3(64, 64), ReLU (block2) MaxPool Conv3(64, 64), InstNorm, ReLU, Conv3(64, 64), ReLU (block3) MaxPool Conv3(64, 64), InstNorm, ReLU, Conv3(64, 64), ReLU (block4) MaxPool Conv3(64, 128), InstNorm, ReLU, Conv3(128, 64), ReLU Unpool Conv3(64, 64), InstNorm, ReLU, Conv3(64, 64), ReLU Unpool Conv3(64, 64), InstNorm, ReLU, Sum block3, Conv3(64, 64), ReLU Unpool Conv3(64, 64), InstNorm, ReLU, Sum block2, Conv3(64, 64), ReLU Unpool Conv3(64, 64), InstNorm, ReLU, Sum block1, Conv3(64, 64), ReLU Conv3(64, 64), InstNorm, ReLU, Sum block0, Conv3(64, 64), ReLU Conv3(64, 24), ReLU), Conv1(24, 2), Sigmoid output: WMH and Cortex masks	Input: a T1 image in the MNI workspace Conv3(1, 12, 3) ReLU, (block0) MaxPool, Conv3(12, 16), ReLU, Conv1(16, 16), ReLU (block1) MaxPool, Conv3(16, 16), ReLU, Conv1(16, 16), ReLU (block2) MaxPool, Conv3(16, 16), ReLU, Conv1(16, 16), ReLU (block3) MaxPool, Conv3(16, 16), ReLU, Conv1(16, 16), ReLU (block4) MaxPool, Conv3(16, 16), ReLU, Conv1(16, 16), InstNorm, ReLU, Unpool, Conv3(16, 16), InstNorm, ReLU, Sum block4, Conv3(16, 16), ReLU Unpool, Conv3(16, 16), InstNorm, ReLU, Sum block3, Conv3(16, 16), ReLU Unpool, Conv3(16, 16), InstNorm, ReLU, Sum block2, Conv3(16, 16), ReLU Unpool, Conv3(16, 16), InstNorm, ReLU, Sum block1 Conv3(16, 12), ReLU Unpool, Conv3(12, 12), ReLU, Sum block0, Conv1(12, 12), ReLU, Conv3(12, 8), ReLU, Conv1(8, 4), Softmask output: Four label maps	Input: a FLAIR image in its native space Conv3(1, 12), InstNorm, ReLU, Conv1(12, 12), ReLU (block0) MaxPool, Conv3(12, 16), ReLU, Conv3(16, 16), ReLU (block1) MaxPool, Conv3(16, 16), ReLU, Conv3(16, 16), ReLU (block2) MaxPool, Conv3(16, 16), ReLU, Conv3(16, 16), ReLU (block3) MaxPool, Conv3(16, 16), ReLU, Conv3(16, 16), ReLU (block4) MaxPool, Conv3(16, 16), ReLU, Conv1(16, 16), InstNorm, ReLU Unpool, Conv3d(16, 16, 3), InstNorm, ReLU, Sum block4 Conv3d(16, 16, 3), ReLU Unpool, Conv3d(16, 16, 3), InstNorm, ReLU, Sum block3, Conv3d(16, 16, 3), ReLU Unpool, Conv3d(16, 16, 3), InstNorm, ReLU, Sum block2, Conv3d(16, 16, 3), ReLU Unpool, Conv3d(16, 16, 3), InstNorm, ReLU, Sum block1, Conv3d(16, 12, 3), ReLU Unpool, Conv3d(12, 12, 3), InstNorm, ReLU, Sum block0, Conv1(12, 12), ReLU, Conv3(12, 8), ReLU, Conv1(8, 3), Sigmoid output: WMH or ROIs, depending on the loaded parameters
		Note: Starting from block 2, Unpool halve dimension in axial planes only

*Note*: All networks are U‐shaped with skip‐connections. Their main difference is the number of convolution kernels, which were hand‐designed following a trade‐off between training speed, model capacity, and resolution. Conv3 and Conv1 refers to the convolution kernel voxel size (3 × 3 × 3 or 1 × 1 × 1). MaxPool operators halve the dimensions and returns indices which are used by Unpool operators.

A set of images where the current segmentation had room for improvement (mainly the false‐negatives with many small lesions missed, or missed lesions close to the cortex, or false‐positive due to bright artifacts), were hand‐selected for further consideration. To assist at this task, and because of the significant number of images remaining (and the lack of a meaningful cut‐off threshold), the samples were displayed on a dedicated data navigator, according to their estimated WMH‐volume in different regions (periventricular, deep white, and infra‐cortical [defined later]), and with an instant feedback feature showing each result as a mosaic of slices, to better appreciate the model success range.

In this process, many images (100 ~ 150), the most outlier ones, were quickly explored. Although a dozen images were dropped due to their segmentation being deemed unrecoverable (e.g., strong motion), 55 images, a number that mostly reflected the amount of effort required, were eventually selected and manually corrected using ITK‐Snap (Yushkevich et al., 2006). The correction used the model output as a starting point. Despite this, we spent, on average 15–20 min on a single image.

The FLAIR ConvNet model was then re‐trained on the curated dataset. The weight attributed to the manually corrected samples was increased by running 10 times more augmentation than the rest of the samples. This second FLAIR ConvNet model was then used to generate the reference FLAIR segmentations, which formed the target of the next step, the T1 ConvNet.

### 2.5 T1 lesions ConvNet model

The T1 ConvNet model, whose role is to segment the WMH lesions in the T1 working space, is the main component of this work. The architecture is a variant of U‐Net, and the parameters are listed in Table 1. One important concern was the resolution gap between the two modalities. While modern T1WI scans are mostly isotropic (~1 mm) 3D images, typical hospital FLAIR sequences produce a relatively small set of thick (~6 mm) but high‐resolution (~0.5 mm) 2D slices. Consequently, beyond the obvious contrast difference between the two modalities, the lesion‐related information also differs in terms of its spatial distribution. In particular, the T1WI image would contain more detailed information in the axial direction than the 2D FLAIR could provide. For cross‐modality learning, while we could simply align the two modalities with an interpolation algorithm, we observed that the final results produced by this method were unsatisfactory. Instead, we used the ConvNet ability to accumulate knowledge across multiple samples in order to supply the missing details of each individual image.

### 2.6 Loss function

**FIGURE 2:**
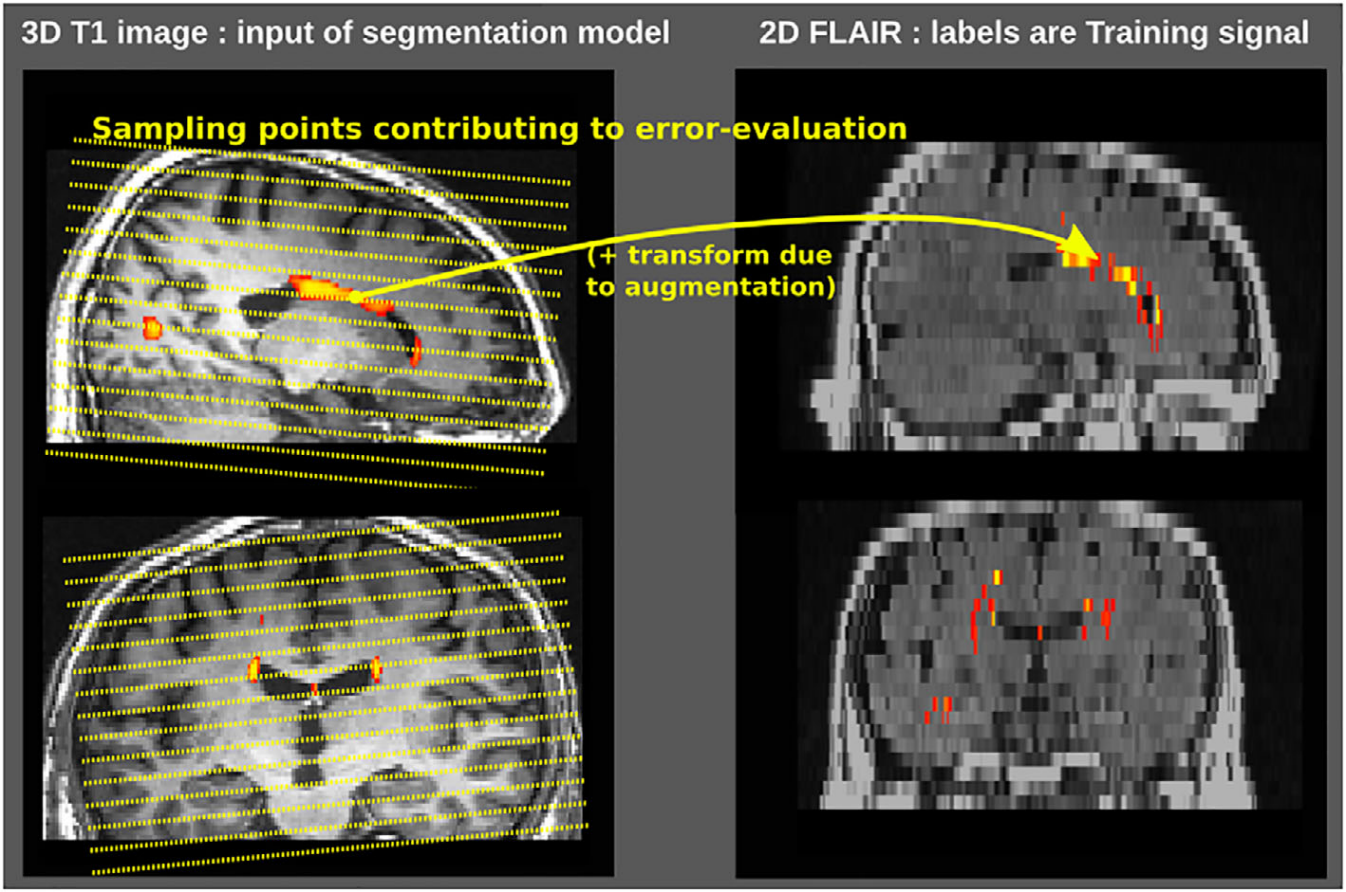
Illustration of the sampling performed during the loss computation. The training signal for each voxel of the target image, in FLAIR space, is back‐projected into a corresponding voxel of the T1 output space, perhaps through an augmentation transform, then back‐propagated further up through the model's convolutional layers. The T1 voxels that are not directly linkable to a FLAIR signal are not affected (in particular, they do not receive an interpolated signal).

To achieve this, we used a spatially sparser loss (Figure 2). The primary output of our network—the WMH mask in T1 space—is dynamically sampled at points where the FLAIR provides the actual training signal, that is, the loss calculation simply ignores all voxels of T1 space that do not map closest to a FLAIR voxel center. This loss is sparse in the sense that each sample contributes only a few slices' worth of training information, less than the higher T1 processing resolution model would optimally need. However, by virtue of being exposed to a large number of subjects, themselves augmented with random affine transforms, the model generalizes naturally from the sparse training information to the full processing resolution.

Second, a T1 lesion model must learn to precisely distinguish subcortical lesions from the cortex itself. Therefore, as an auxiliary helper task, we explicitly trained the model to produce a cortical ribbon mask as a secondary output. This was performed entirely in the T1 input space. From previous experience, this is a relatively easy task in terms of required ConvNet model capacity and should not, therefore, hinder the primary outcome. Besides providing a supplemental, explicit training signal, this helped us to understand the behavior of the model during its optimization.

We used a Euclidean distance in both parts. We favor this simple metric as it can easily be interpreted in terms of an independent voxel probability. The final loss function is thus the weighted sum of the sparse FLAIR lesion signal and the cortical segmentation loss:

$$L=\sum_{\left(\text{FLAIR}\right)}{\left(S\left({\text{out}}_{1}\right)-\text{FLAIR}\_\mathrm{wmh}\right)}^{2}+0.001\times \sum_{\left(T1\right)}{\left({\text{out}}_{2}-T1\_ribbon\right)}^{2},$$

where the out_*i*_ are the model outputs in T1 space; *S*: T1 → FLAIR is the voxel sampling function; FLAIR_wmh and T1_ribbon are, respectively, the target lesion and ribbon masks, in their own FLAIR and T1 spaces. The FLAIR_wmh masks are the one obtained from the previous step (the FLAIR‐ConvNet step), while the T1_ribbon masks are simply FreeSurfer cortical segmentations.

The training set was made from 4096 subjects (and included the subjects used to train the FLAIR ConvNet). In order to augment the T1 dataset, and in addition to the random affine transformations, we randomly altered the image histogram to simulate the variation of contrast of different scanners and sequences.

### 2.7 Regions of interest

**FIGURE 3:**
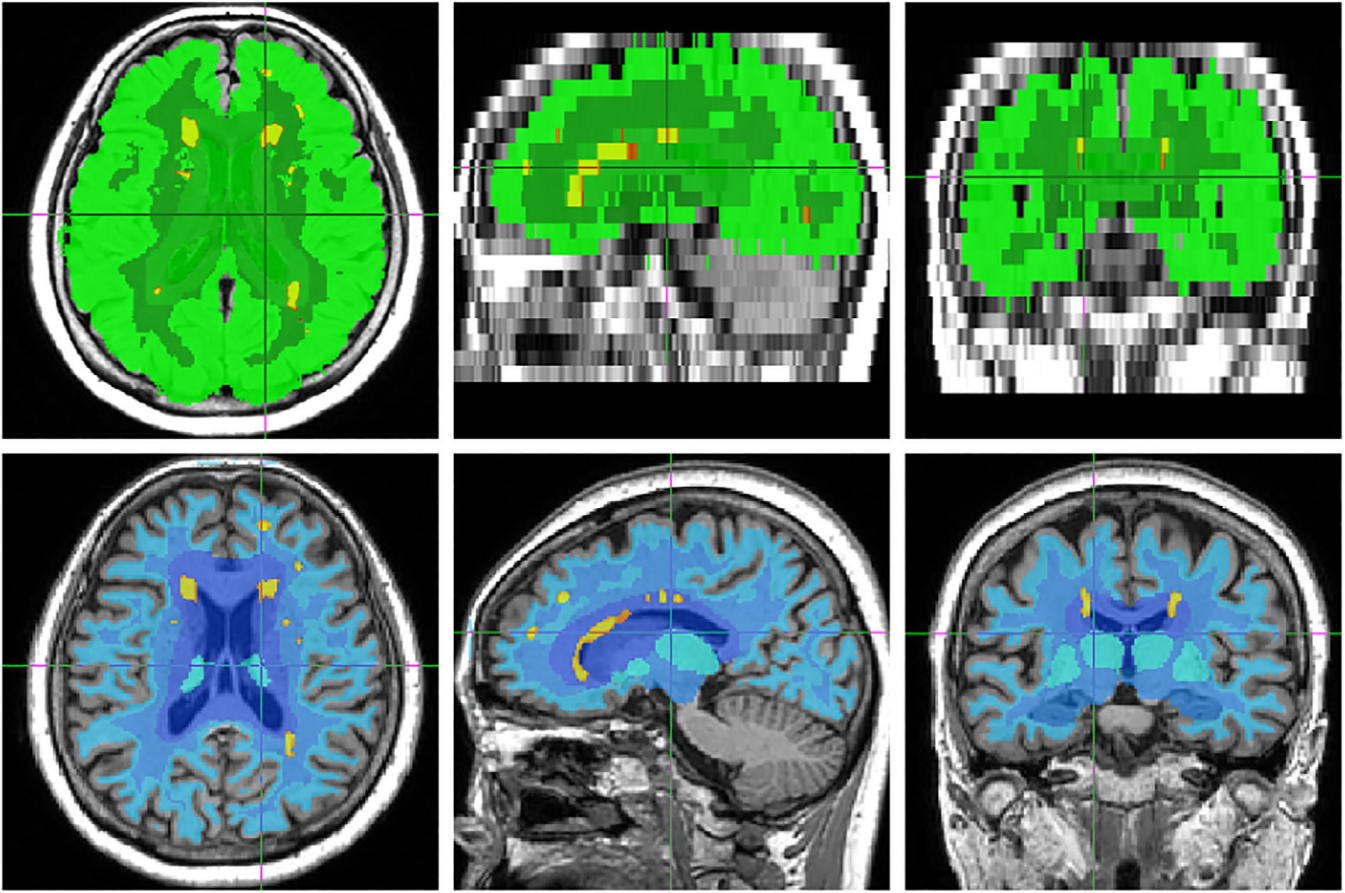
Two separate ConvNets were tasked with generating relevant regions of interest (ROIs) represented as color shades for 2D FLAIR (top, greens) and for T1 images (bottom, blues). The FLAIR model, used for temporary usage, was only approximate because of the poor resolution and lack of clearly visible structures. The final model, on T1, was intended to classify lesions (identified and depicted as yellow patches). Still, the hard borders of the ROIs make the current aggregation method perfectible.

A separate ConvNet model was created to quickly identify the main regions of interest for WMH, based on prior knowledge. This region set served two purposes. First, to organize the dataset in terms of lesion load, to ease data exploration, and to spot outliers or other unusual cases of interest. Second, to create the final, clinically relevant summary of lesion distribution. Since these two purposes applied, respectively, to the FLAIR and the T1 modality, which have different constraints, two separate models were actually trained (Figure 3), because we would not benefit from having a single multi‐modal model (Zopes et al., 2021) here.

We aimed to follow the common practice and guidelines (Thompson et al., 2018), which separate the WMH in periventricular, deep white, or juxtacortical (infra‐cortical) lesions. For the periventricular ROIs, we used a 9 mm‐expanded mask of the ventricles in MNI space, a somewhat arbitrary visually defined limit. We noted that (Coupé et al., 2018) used 3 mm in MNI space but also observed that this failed to adequately cover the periventricular lesions for a large number of our elderly subjects. Nevertheless, on a Fazekas scoring experiment, the PWML (periventricular) class was the most inconsistently rated, which suggests that a simple ROI approach may never be fully satisfactory.

The ROIs were generated using an initial combination of FreeSurfer label masking, modality co‐registrations, and morphometric algorithms—all of which were relatively slow. This initial outcome was then itself learned by two dedicated ConvNet, one per modality, using 4096 subjects. This not only benefited the eventual runtime speed but also allowed to recover from algorithmic failures that may occur during the ROI creation process.

One problem worth mentioning is that FreeSurfer, or other similar tools, may sometimes get confused about pathologies in the white matter, and may not explicitly label them as white matter because of their unusual intensities, even when relying on spatial priors. However, for our purpose, the ROIs should cover the whole white matter, without any holes. Furthermore, it is also known that some WML can evolve into lacunar infarcts (de Jong et al., 2002; Gouw et al., 2008). Therefore, to overcome this systematic source of error, our T1‐ROI model was trained on images with simulated lacunes that were generated by randomly scattering the white matter of the input images with small dark‐intensity patches.

### 2.8 Evaluation

To evaluate the trained model, we first ran a large scale analysis on the training set. We compare the mask of lesions estimated from T1W images against the LST‐estimated lesions from the FLAIR images, computing the DICE coefficient as a measure of overlap. Second, to give a clinical perspective, a manual expert rating is conducted on a subset of 200 test image, using two expert raters.

### 2.9 Computational tools

Most of the spatial transformations and image processing were computed using ANTs (Avants et al., 2009), although the modality co‐registration was conducted with NiftyReg. We also used the c3d tool from ITK‐Snap. The MRIs were processed on FreeSurfer 7.1 on four multi‐core machines, while the FLAIR images were processed using LST on MATLAB. The various analyses and image processing scripts relied on Python 3, NiBabel, and the SciPy stack. The data navigators and rating interface relied on Matplotlib and other web technologies. PyTorch (1.6 then 1.8) was used for the implementation of ConvNet, conducted on an NVidia RTX 2048 GPU using the ADAM optimizer.

## 3 RESULTS

### 3.1 Qualitative example

**FIGURE 4:**
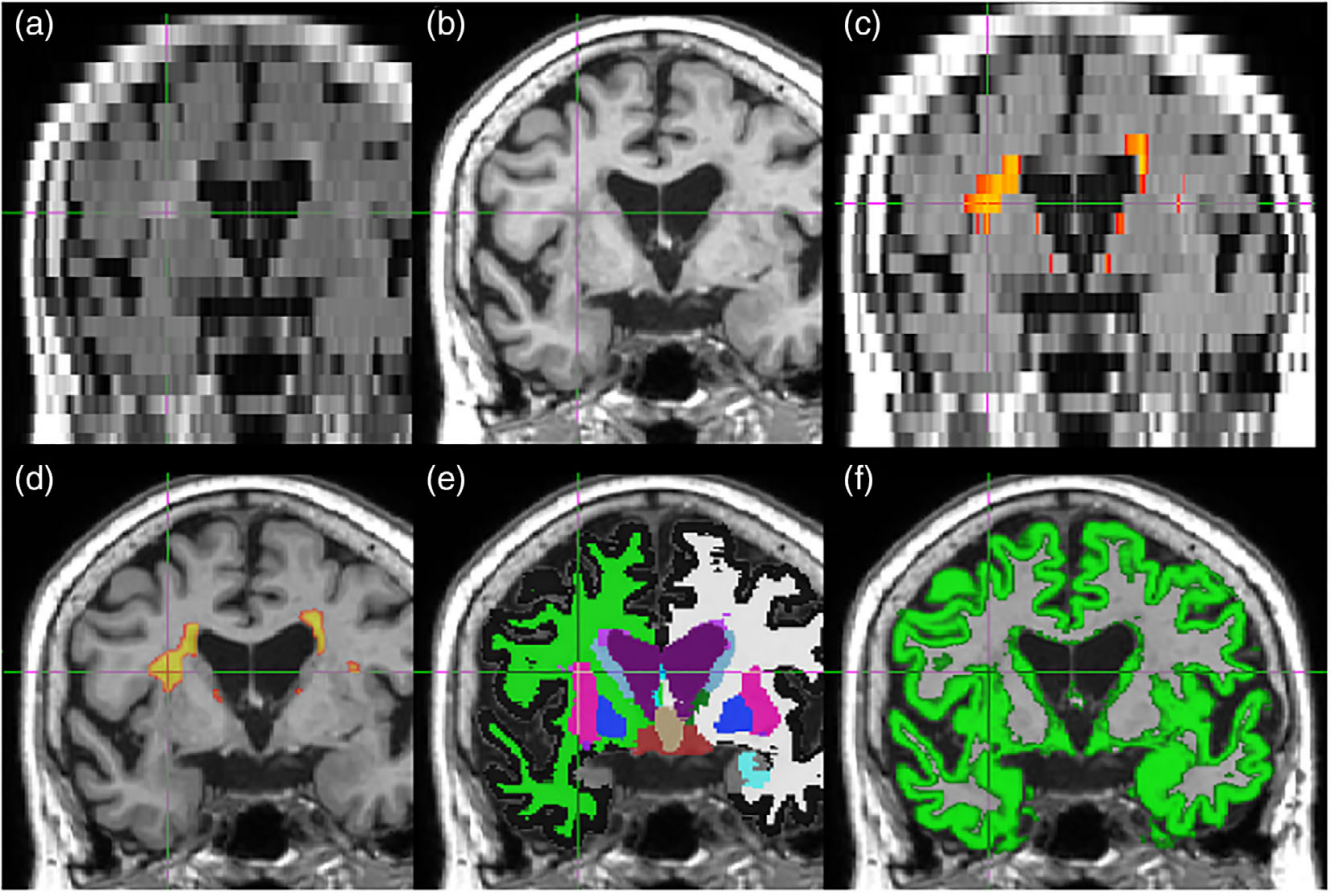
Methods overview. On this coronal slice, a periventricular lesion expands toward the insular cortex. The lesion is visible on the original 2D‐FLAIR image (a), and on the higher‐resolution T1 image (b) although with less contrast. The LST algorithm outcome (c) correctly identified the lesion on the FLAIR image. Our trained ConvNet successfully identified a similar area (d), although at the higher T1 resolution, despite the contrast ambiguity. However, FreeSurfer (e) misclassified part of the lesion as the putamen, while SPM (f) wrongly included it in its gray‐matter mask.

Figure 4 depicts a single slice of MRI images of a random participant under various conditions. The resolution gap between the FLAIR and T1 sequences is clearly visible on this coronal view. The contrast between the lesion and the gray matter is much lower on the T1, which explains why it can be difficult for both people and classic image processing algorithms to identify it accurately.

### 3.2 Effect of the loss function

**FIGURE 5:**
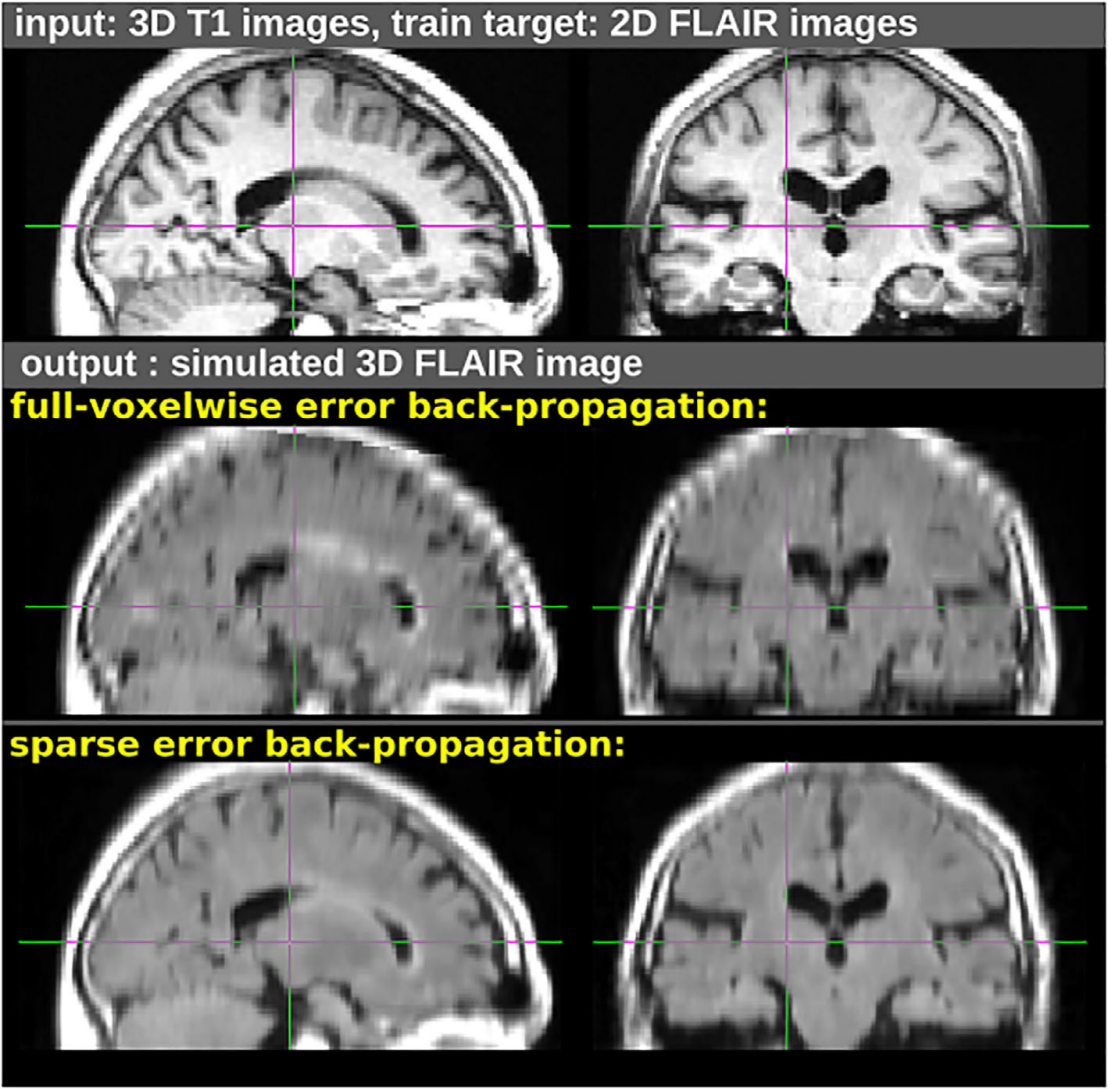
Illustration of the effect of two loss functions, used here for a cross‐contrast transformation experiment. In this example, a ConvNet was trained to change an input T1‐weighted image (top) into its corresponding FLAIR contrast, based on 2048 training pairs. The MRI sequences used for the target FLAIR contrast have a lower native vertical resolution. Using a standard voxelwise loss function (middle), the model over‐learned the interpolation artifacts. However, using the spatially sparse feedback‐signal loss (bottom), the model successfully learned the contrast transform while maintaining the resolution.

Figure 5 illustrates the effect of the sparse feedback on the resolution, using a simple contrast‐transfer task. In this experiment, a ConvNet must produce a fake image of FLAIR contrast corresponding to an input T1 image. The training is conducted using pairs of high‐resolution 3D T1 images and 2D FLAIR images, using either a standard voxelwise interpolating loss or a spatially sparse loss. Once trained, visual inspection reveals that while the former appears to learn spurious aliasing artifacts, the latter can preserve more spatial features of the input T1 along the axial axis.

### 3.3 Systematic automated comparison

We ran a large‐scale automated comparison against FreeSurfer, using the FLAIR image as a reference. The lesion ConvNet was trained on 4096 subjects, and the 3598 remaining images comprised the test set. Each T1 image in the test set was analyzed for WMH by both FreeSurfer 7.1 and our ConvNet. Next, the segmentation masks of both methods were resampled into the original FLAIR resolution and a DICE metric computed against the LST‐segmented FLAIR. We thresholded the probabilistic maps at 50%, although the actual threshold value did not significantly alter the overall result.

**FIGURE 6:**
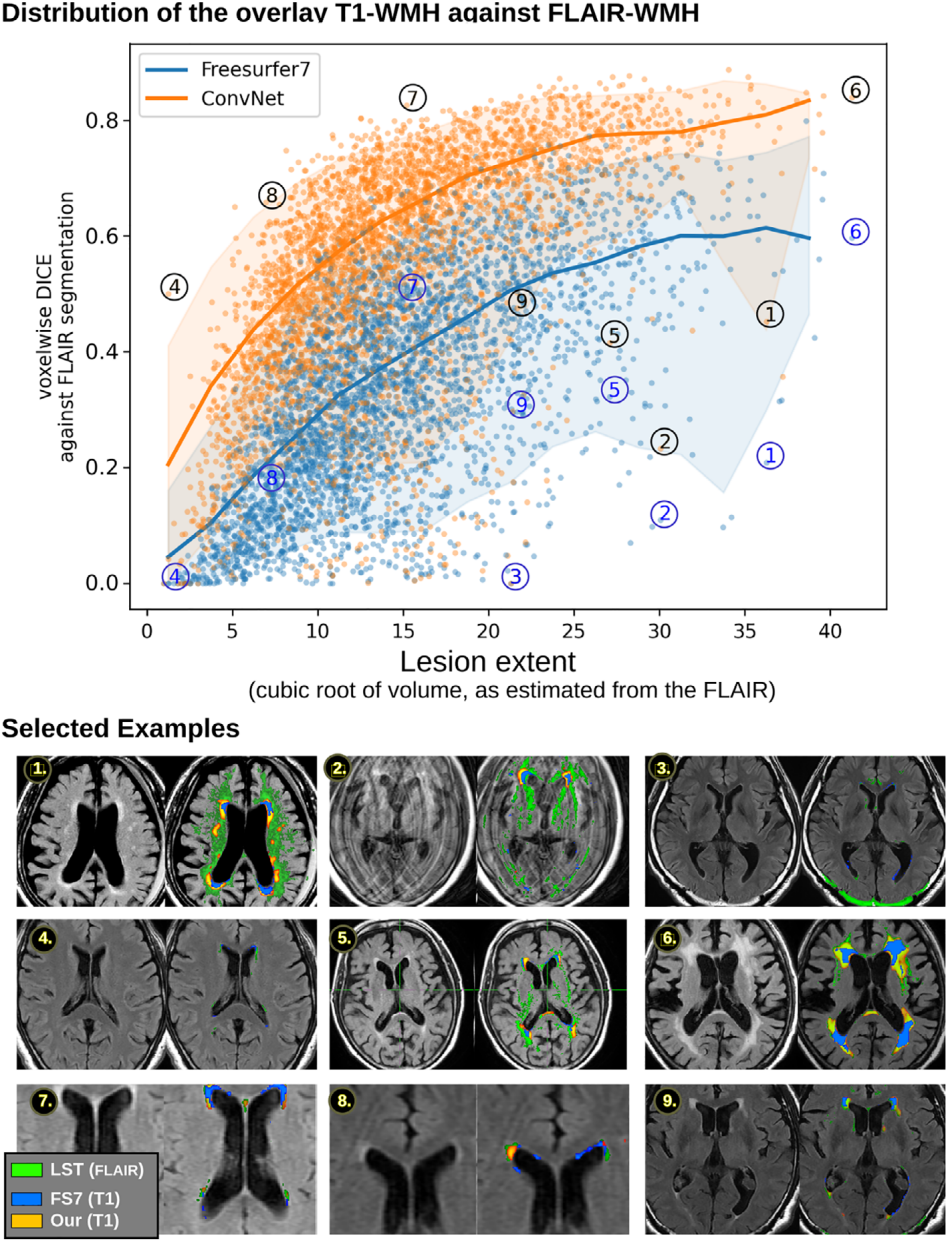
Agreement between the T1‐based segmentation methods and the FLAIR segmentation method (top). The graph shows the distributions of the overlap as a DICE metric calculated in the resampled FLAIR space and ordered by lesion volume. Colors encode the two methods, and the filled areas contain 90% of the subjects at each volume bin. The mean/SD DICE is 0.33/0.18 (FS7) and 0.55/0.19 (our). Selected MRI cases (bottom), corresponding to numbers on the plot, are selected to illustrate different occurring patterns of overlap.

Figure 6 shows the DICE distribution for each method with the samples ordered by lesion volume. We note that the overall DICE metrics are low, particularly for small lesion loads, but this does not preclude us from using the metric for large‐scale observation purposes. This is due to the transfer‐learning across modalities, which reveals the difficulty of precisely locating the lesions and agreeing on their boundaries in true anatomical space. Our ConvNet segmentation was consistently better than FreeSurfer 7 (*paired*‐*t*__[__3598__]_ = 116.0, *p =* 0), although 1.63% of the test set showed a better DICE against LST for FreeSurfer. Naturally, part of ConvNet's success can be attributed to its more explicitly chosen goal of segmenting a T1 in a way that resembles a FLAIR LST delineation.

A selection of extreme points are highlighted to illustrate the variety of patterns that can occur. Low DICE values may be attributed to the failure to segment FLAIR itself due to motion or intensity threshold (e.g., top row). Other causes of low DICE were due to the plain lack of lesions, where pixel‐perfect segmentation is rarely achieved. The best DICE scores were obtained when the lesion load was heavy, although those cases are not necessarily the most relevant from a clinical perspective, since it is often already too late for the subject to positively alter the course of WMH evolution.

### 3.4 Human visual rating

While certainly useful, the lesion mask obtained from using LST on FLAIR also has limitations, even when disregarding putative cases of algorithmic errors. To pursue the evaluation further, a random subset of the dataset was selected for a visual investigation.

In order to submit a relatively large sample to visual judgment, we designed a web rating user interface that enabled a three‐way comparison between pictures of the three methods and allowed the expert to pick the best and worst proposal, in a double‐blinded setup.

We found that such a system of comparison was more suitable than an absolute‐scale rating requirement, which proved hard to keep consistent across a large number of subjects, let alone across raters. This also proved more reasonable in terms of the effort required by the human experts, than an alternative like producing manual segmentations.

A set of 200 subjects were randomly selected from the test set, and pictures of each segmentation (ConvNet, FS7, and original LST) were generated, thresholding the masks when appropriate (LST and ConvNet). The model outcome was reviewed by two certificated radiologists (Y. Tat and L. C) over their original FLAIR slices. This had the following advantages: the expert raters were already used to inspecting FLAIR image rather than 3D T1; images; the FLAIR images had fewer axial slices and therefore needed less visual effort to review a subject; and some interpolation artifacts could have otherwise revealed which image comes from the FLAIR space (i.e., LST method), thereby partially nullifying the double‐blind setup.

**FIGURE 7:**
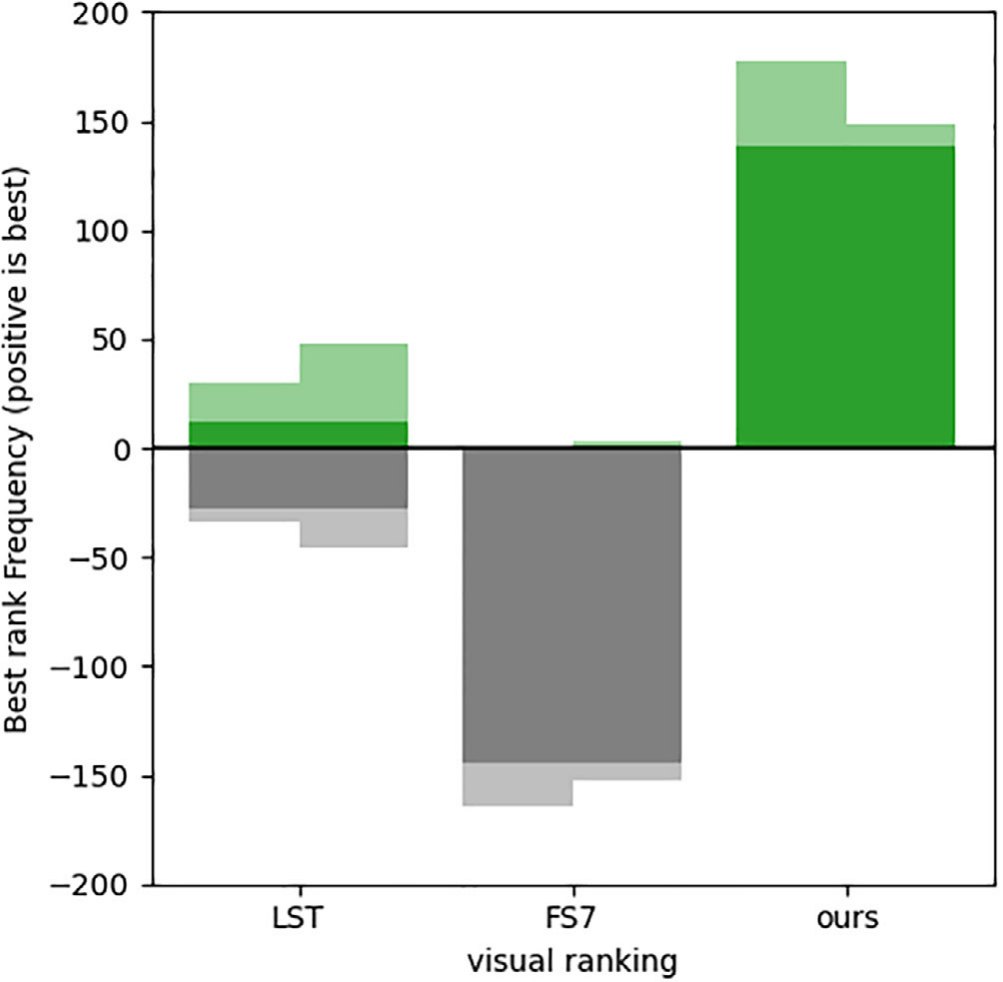
Results of the human expert evaluation. The bar chart shows the number of times the method result was considered as the best (or, for negative, worst) by the two raters (sub‐columns). The darkest areas represent segmentations where the two raters' judgments agreed. The results may not sum exactly to 200 as ties were allowed, albeit discouraged.

The two human raters independently rated the same 200 subjects. Figure 7 depicts the number of times each method was ranked the best. Some differences were observed among the two raters (*χ* ^*2*^ = 7.53, *p =* 0.023). When raters agreed on a positive rating (152 out of 200 subjects), 91% were in favor of our ConvNet. Furthermore, when selecting LST as the best segmentation, their ratings were unanimous for only 18% of the images, revealing the role of subjectivity in that choice. Finally, the raters largely agreed that the FreeSurfer segmentations were generally less accurate, at least in our setup. These method choices appeared to be irrespective of the lesion volume (i.e., the rater choices were insignificant as predictors of the volume, *F_*_[__3196__]_ = 0.85, *p =* 0.46), even though one rater reported that the rating task was easier and faster for images with larger lesion loads.

## 4 DISCUSSION

We endeavored to train a ConvNet to extract WMH from the T1 images. Obtaining a ground truth was not envisionable beyond a small sample due to the large annotation effort required (and the lack of such public datasets, with a compatible license). Instead, as a starting point, we used cross‐modality learning to benefit from the algorithms that already existed for FLAIR images. A ConvNet was trained to learn the dataset, and some of the most difficult cases, selected by an informed‐guess strategy, were hand‐fixed and returned back to the training loop. Next, those cleaned masks of FLAIR space were used as targets for the training of our main T1W ConvNet across contrasts, using a loss function intended to overcome the resolution difference. We evaluated the results with an automated region‐overlap comparison against other methods, then with expert rating.

The lack of ground truth was not only a technical matter, however, as we observed disagreement among the experts related to qualitative description of the lesions. In fact, on a Fazekas rating effort of 200 subjects from a single site, two experts from different hospitals obtained an agreement (same rank on the 4‐level qualitative scale) of only 62.6% for deep‐white lesions, and as low as 38% for the periventricular lesions. Although reportedly not always necessary (Boutet et al., 2016), our results suggest the need for some preliminary coordination before using the scale. In the design of our visual evaluations, the two raters largely agreed across an equally sized set of 200 subjects.

### 4.1 The use of T1 images

In this study, we targeted T1‐weighted images. WMH lesions had been associated, with various levels of confidence, with multiple brain pathologies. Therefore, it is useful to access this information using more than the FLAIR modality alone, particularly in the context of cohort research, which often acquires a T1 image for structure localization and measurement purpose. In fact, the JPSC‐AD large‐cohort project is such a case of a project that did not enforce a FLAIR acquisition at all the participating sites. As another example, (Tanaka et al., 2021) studied a large cohort of various ages, but only acquired T1 images rather than FLAIR.

Additionally, (Wei, Poirion, et al., 2019; Wei, Tran, et al., 2019) compared the predictive power of both T1 and FLAIR lesions—respectively segmented with FreeSurfer 6 and SPM‐LST—and concluded a “general equivalence between these two,” based on a similar association with abnormal β‐amyloid and tau, although other researchers (Hotz et al., 2021) assessed that WMH lesions labeled by FreeSurfer v6.0.1 are no substitute for manual effort. Still, a T1 image provides potentially more spatial precision, which could prove useful for accurate longitudinal monitoring of early smaller lesions, particularly with tailored methods (Sudre et al., 2017).

### 4.2 Cross‐domain learning

Initially, we tried to work in T1 space directly, by using the masks generated by FreeSurfer as a training signal. Unfortunately, this method turned out to be too unreliable, despite the ability of ConvNet to be robust against occasional mislabeling in the training set (Thyreau & Taki, 2020). We observed that the model initially learned to segment a majority of the WMH, and then, instead of refining its learning, appeared to unlearn whole classes of lesions as more training iterations progressed and more inconsistent samples were presented. We had to cease pursuing this initially attractive approach of copying FreeSurfer T1‐processed images, and instead developed the new cross‐contrast learning model described here.

We proposed a transfer‐learning approach where the set of 2D FLAIR images would provide sufficient information to train a T1 classifier. A spatially sparse loss mostly solved the resolution issue. The reason for this was because even though each image contains little information individually, the whole training set was large. Such a spatially sparse feedback loss is suitable for any similar cross‐modality learning setup where a super‐resolution approach of the target is required, due to images having widely different resolutions, by themselves or by the corresponding labels (e.g., to reduce the annotation effort). Learning from sparser annotations is often a worthwhile goal in the field of medical image segmentation.

### 4.3 ConvNets for the WMH lesions

The ConvNet research work has often focused on the small sample regime and architecture improvement (Fartaria et al., 2016; Ghafoorian et al., 2017; Guerrero et al., 2018; La Rosa et al., 2020; Li et al., 2018; Li et al., 2021; Liang et al., 2021; Moeskops et al., 2018; Orbes‐Arteaga et al., 2018; Valverde et al., 2017). Recently (Kuijf et al., 2019) organized a competition, using 60 (3D or thin‐sliced 2D) FLAIR images as training set, and 110 manually labeled subjects for evaluation. Here again, the focus was squarely on the ability of the method to generalize from the provided sample. Notably, ConvNets ranked favorably, although the organizers pointed that that three‐dimensional ConvNets achieved lower‐ranking results, which they attributed to the 2D nature of FLAIR images. They also noted that the moderate recall of the individual observers is mainly caused by either not segmenting or missing small WMHs (Kuijf et al., 2019). We hope that the current work improved on that situation.

Finally, we note that ConvNets provide attractive runtime properties. They offer robust results with a constant‐time inference. Our current implementation requires around 20 s to fully process a T1 image, using four threads of a recent CPU and can consume up to 4Gb peak memory. However, the network topology of our model was partly defined by a practical concern: the maximum size that could allow a single sample to fit in the GPU memory for a forward and a backward pass, at the useful input resolution of 1 × 1 × 1.5 mm, and with the added cortical‐ribbon segmentation requirement. This is because we wanted to focus on the dataset learning behavior, without worrying about additional failures attributable to a more limited model capacity. A logical future work would aim to reduce the model size, using one of the many model reduction techniques proposed in the field.

### 4.4 Limitations of the final model

To validate our model, we ran an automated evaluation based on the similarity with FLAIR masks, and compared it to the FreeSurfer 7 model. There are some limitations in this approach. The DICE coefficient was conducted in FLAIR space, considered as a rough ground‐truth, and therefore, would not capture the fine lesion details along the slice axis. And as mentioned above, the FreeSurfer 7 output was not always of satisfying quality. Importantly, we observed that the DICE value was generally higher when the lesion volume was bigger. This phenomenon is in part because, in the DICE computation, the relative impact of each missed voxel is stronger for small segmentations, but also because the numerous smaller lesions are less consensual over different methods, or even different human ratings. To highlight this limitation, we reported the DICE coefficients as a function of the lesion load.

The visual evaluation showed segmentation in FLAIR space even when created from T1 images. Therefore, the accuracy was not judged explicitly in the axial direction. The segmentation quality would probably be evaluated even more favorably when seen through coronal or sagittal slices; however, we aimed for a clinically realistic comparison. Overall, the limited axial resolution in many FLAIR sequences is generally not perceived as a major hindrance for the descriptive purposes of clinical reviews. It becomes a difficulty only when dealing with quantification. Some successful quantitative algorithms of FLAIR images actually rely on an additional structural image to compensate for the loss of localization power, and they are generally only used for research.

Finally, we note that the lesion delineation is necessarily sensitive to the source image contrast and the threshold chosen, as often with MRI image quantitative analyses. Our ConvNet has been trained on the variety of scanner of the JPSC‐AD cohort, and should therefore generalize to most T1 images (see also Appendix S1 for an application over the OASIS 3 dataset [(LaMontagne et al., 2019]); However, should high accuracy be required, the lesion volume should not be compared between different contrasts without care, as the actual absolute volume value may impacted by the acquisition sequence.

## 5 CONCLUSION

In this manuscript, we described the training procedure of a ConvNet whose goal was to segment WMHs from T1 images, and which constitutes the core component of a corresponding end‐user software, which is available for download. The training of the T1 model had been achieved by transferring some information that is more reliably found in FLAIR images, enhancing it using a limited amount of human guidance, and making adjustments to the loss function. The large JPSC‐AD dataset used as training material was key to the robustness. This study provides another good example of the power of ConvNet from the perspective of working across domains to solve problems more optimally.

## AUTHOR CONTRIBUTIONS

Benjamin Thyreau and Yasuko Tatewaki designed research; Benjamin Thyreau developed the method; Yasuko Tatewaki, Liying Chen analyzed the images; Yasuko Tatewaki, Liying Chen, Naoki Hirabayashi, Yoshihiko Furuta and Jun Hata performed clinical evaluation; Yuji Takano, Naoki Hirabayashi, Yoshihiko Furuta, Jun Hata, Shigeyuki Nakaji, Tetsuya Maeda, Moeko Noguchi‐Shinohara, Masaru Mimura, Kenji Nakashima, Takaaki Mori, Minoru Takebayashi collected data; Yasuyuki Taki and Toshiharu Ninomiya coordinated the project; Benjamin Thyreau, Yasuko Tatewaki and Liying Chen wrote the paper with inputs from all authors.

## FUNDING INFORMATION

The JPSC‐AD study was supported by the Japan Agency for Medical Research and Development (JP21dk0207053) and Suntory Holdings Limited (Osaka, Japan). The funders had no role in the design of the study, the collection, analysis, and interpretation of data, or the writing of the manuscript.

## CONFLICT OF INTEREST

The authors declare no conflict of interest.

## ETHICS STATEMENT

JPSC‐AD was first approved by the Kyushu University Institutional Review Board and then by the ethics committee of each research institute. All participants provided written informed consent for baseline and follow‐up surveys.

## Supporting information


**APPENDIX S1** Supporting Information

## Data Availability

The trained model available for download at https://github.com/bthyreau/deep-T1-WMH. The datasets generated and/or analyzed during the current study are not publicly available due to restrictions included in the informed consent of research participants. However, data are available from the authors upon reasonable request and with the permission of the Japan Agency for Medical Research and Development.
