# Supplementary material for: Higher‐resolution quantification of white matter hypointensities by large‐scale transfer learning from 2D images on the JPSC‐AD cohort
Source: Hum Brain Mapp. 2022 May 7;43(13):3998–4012. doi: 10.1002/hbm.25899 (PMC9374893; doi:10.1002/hbm.25899)
Supplement: Supplementary file 1 — APPENDIX S1 Supporting Information [file HBM-43-3998-s001.pdf]

Supplementary Information

1. Some failure cases of the initial LST algorithm

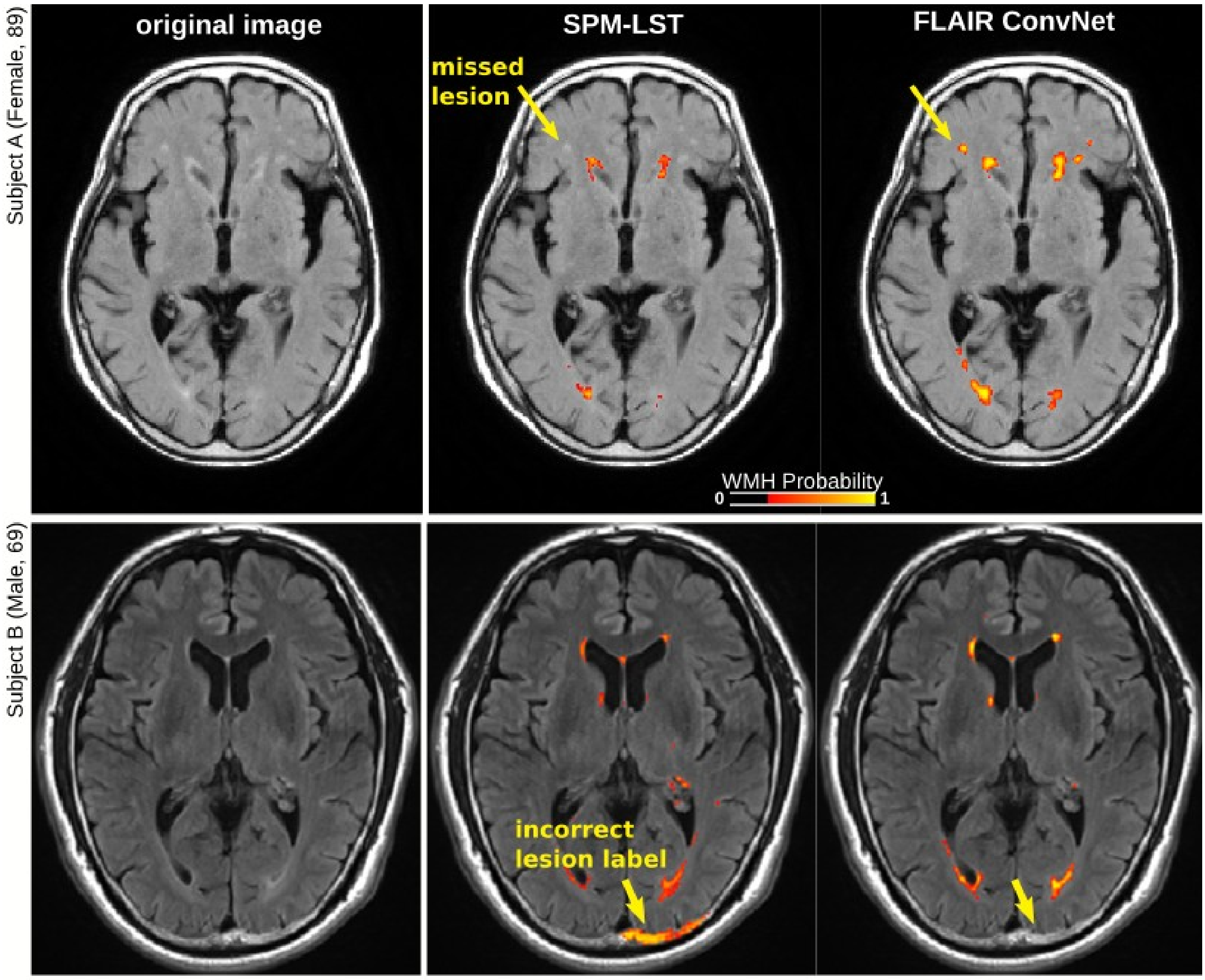

Fig S1. Example of FLAIR images where the LST algorithm appeared suboptimal, but where the FLAIR ConvNet improved the segmentation. Two selected cases (rows) of FLAIR with low WMH load are depicted, as original image, SPM-LST, and FLAIR ConvNet masks. The arrows point to specific areas of improvement.

2. Manual Improvemement in the FLAIR model.

During development of the FLAIR model, we used a semi-automated method to look for failure or suboptimal cases, and a subset of subjects were selected for manually improvement.

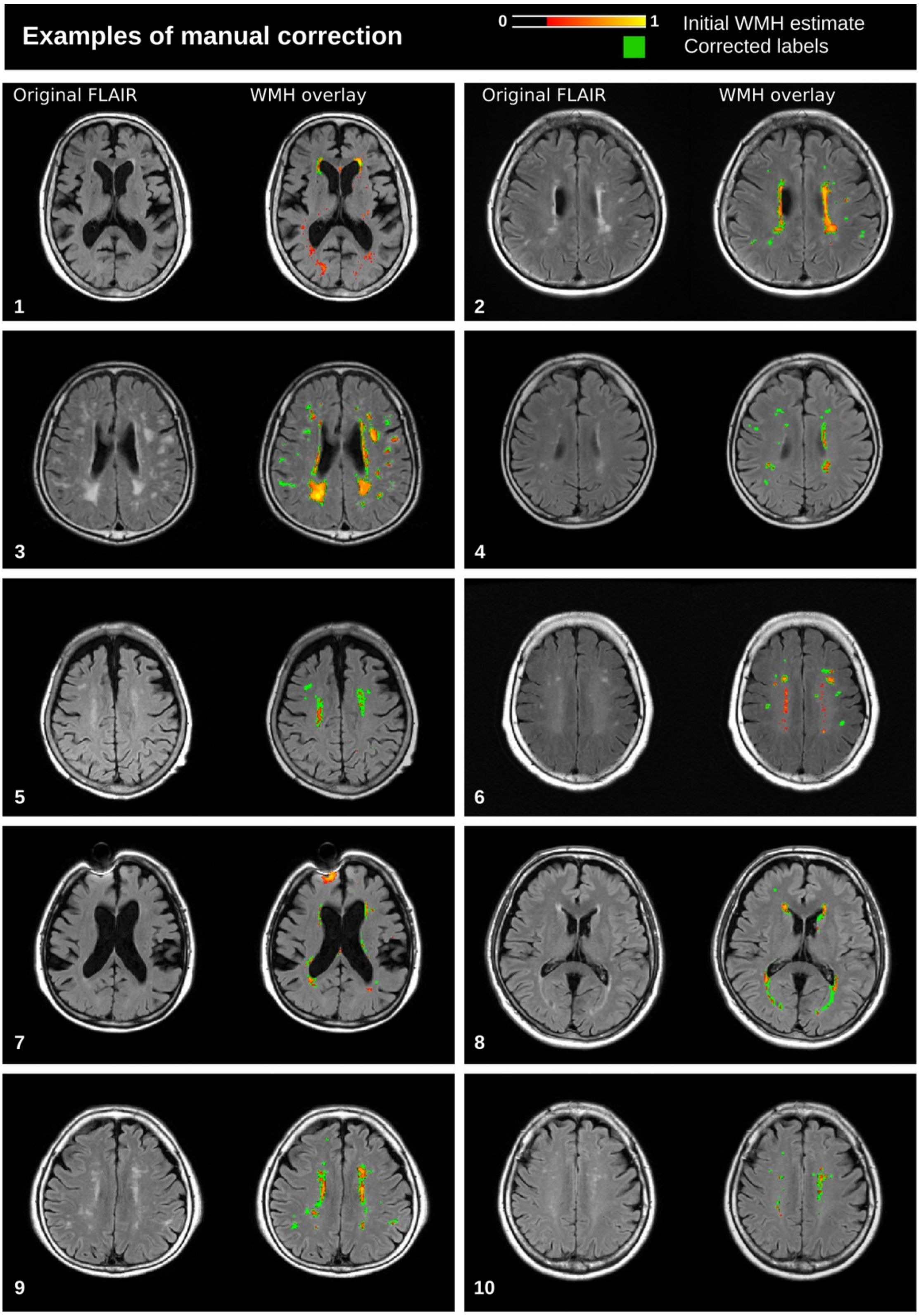

Fig S2.1 A FLAIR slice for ten random subjects (out of the 55) that we manually fixed. The probability map (red-orange) shows the original LST-software prediction, whereas the green masks is the manual segmentation. We noted various erros patterns, such as False-Positive segmentation (e.g. Cases 1, 6), Artefacts (Case 7), missed punctuate lesions (several cases) or lesions very close to cortex (Cases 3, 9)

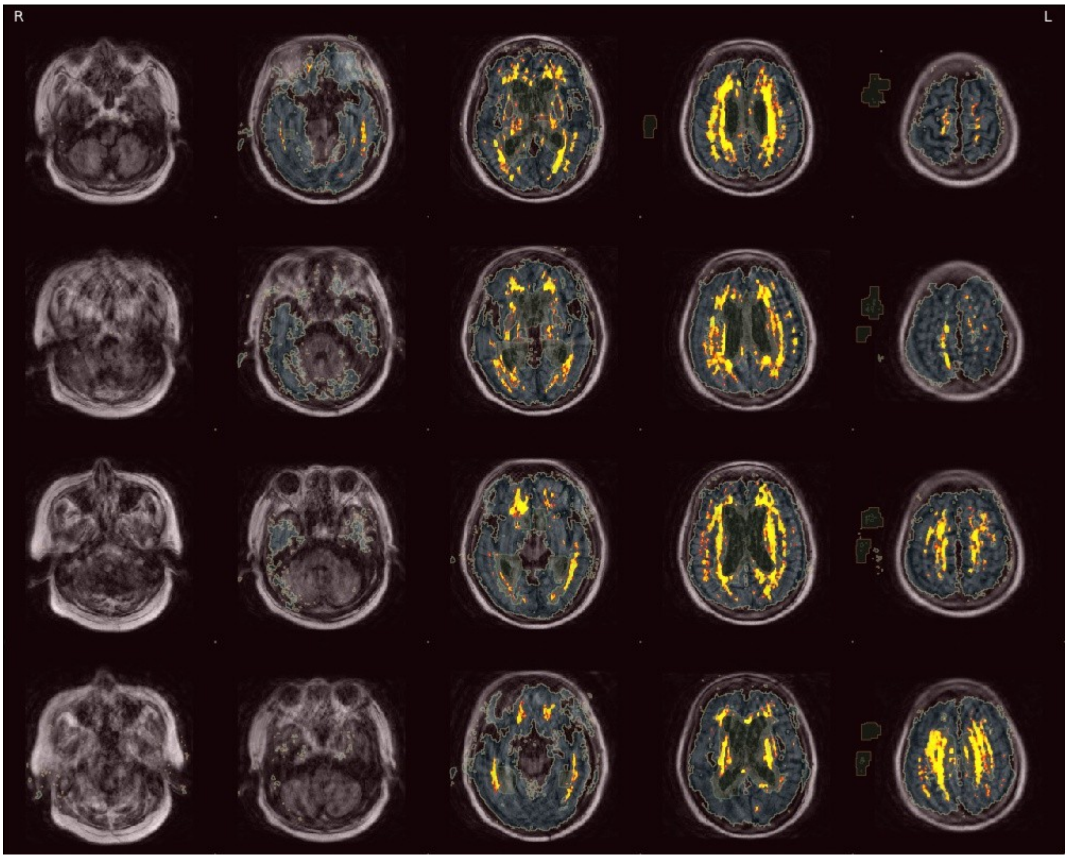

Fig S2.2 An example of subject dropped from further analysis. This subject was noticed during the visual data exploration, and we believed this image was not worth fixing and would not bring any good training signal to the model. Red-Yellow shows the candidate segmentation. The muted color area are the estimated Region of Interet.

3. Application on the OASIS Dataset

We ran an additional experiment to illustrate the application of our trained ConvNet on a completely independent sample using the OASIS 3 cohort, a NeuroImaging dataset focused on normal aging and Alzheimer Disease (*La Montagne et al, 2019*). We downloaded all FLAIR images available on the OASIS 3 project xnat server, and the corresponding T1W images. This way, we obtained pairs of images for 606 subjects. The FLAIR sequences had 35 slices, which is thinner than the 2D hospital FLAIR that were used in our manuscript. For simplicity, we did not perform any co-registration, as it seems that the FLAIR were already well aligned to the T1W.

We applied our ConvNet over all T1W images, and ran the LST algorithm on the FLAIR images. Then, we computed a DICE coefficient in the FLAIR image space by resampling each T1W WMH resulting mask onto its corresponding FLAIR image. Fig S3. (next page) shows the distribution of the DICE coefficient (with mean, and quantiles). Similar to our own JPSC-AD testing-set, the DICE metric improves with the overlap size. A selection of case if depicted for qualitative illustration.

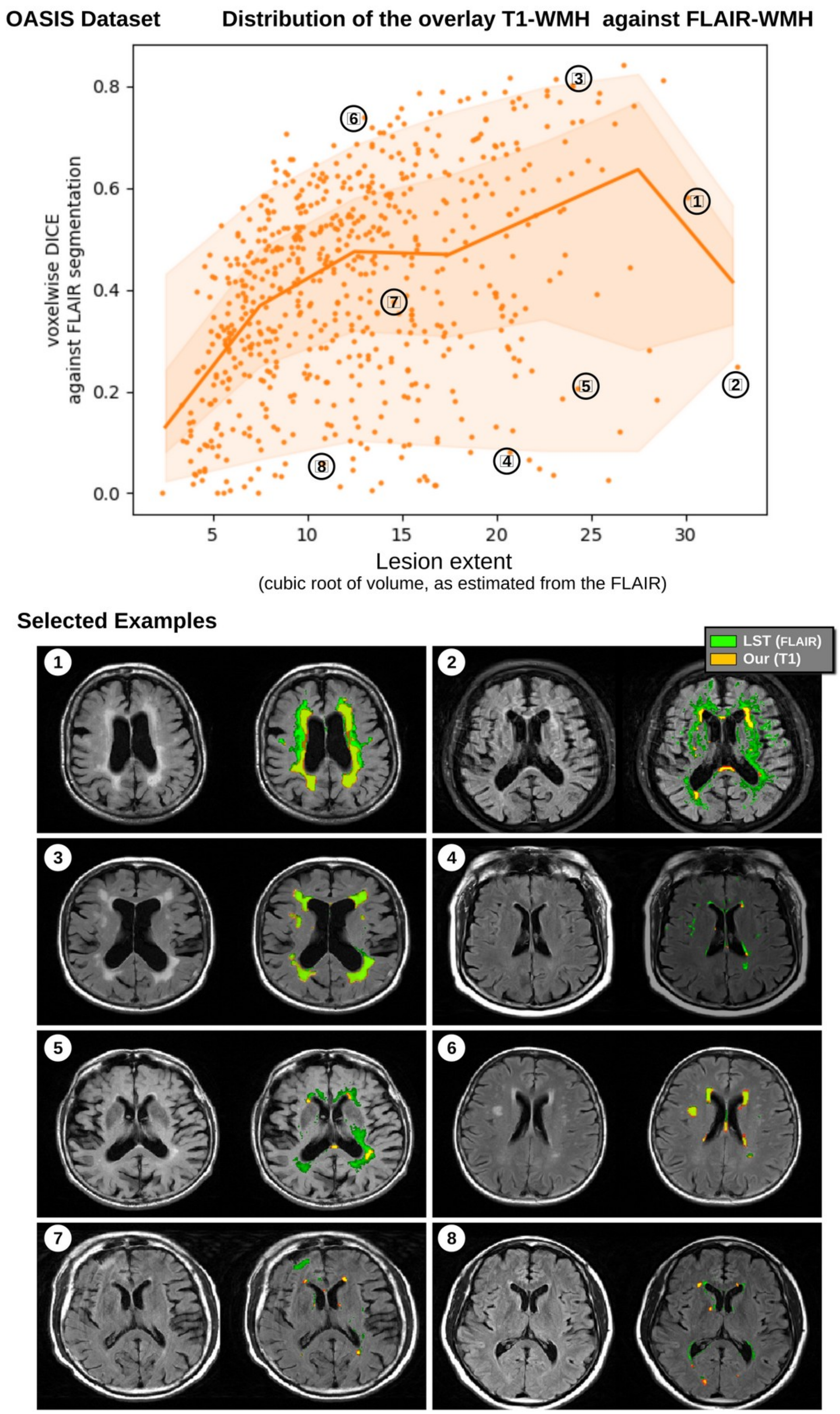

Fig S3. Distribution of DICE for FLAIR-LST vs T1-WMH ConvNet, in FLAIR space; OASIS dataset, 606 subjects. The DICE coefficient (mean/sd: 0.41/0.2) is shown as a function of lesion load (color lines for 0.5, 0.75 and .95 quantiles). A selection of cases is depicted, including some with good agreement (Cases 3, 6, 1), but also false-positive segmentation (Case 4, 5), alignement failure (Case 8), motion (Case 2), and even a distortion artefact which resemble a WMH (Case 7), which explain some of the lower DICE scores.
